# Comparative analysis of antimicrobial resistance and genetic diversity of *Bordetella bronchiseptica* isolates obtained from swine within the United States

**DOI:** 10.3389/fmicb.2024.1501373

**Published:** 2024-11-28

**Authors:** Tracy L. Nicholson, Sarah M. Shore

**Affiliations:** National Animal Disease Center, Agricultural Research Service, United States Department of Agriculture, Ames, IA, United States

**Keywords:** *Bordetella bronchiseptica*, whole-genome sequencing (WGS), virulence factor, average nucleotide identity (ANI), sequence type (ST), antimicrobial resistance (AMR), mobile genetic element (MGE), swine

## Abstract

**Introduction:**

*Bordetella bronchiseptica* is bacterial pathogen that is pervasive in swine populations and serves multiple roles in respiratory disease.

**Methods:**

This study utilized whole-genome sequencing (WGS) analysis to assess the sequence type (ST), identify the genetic diversity of genes predicted to encode regulatory and virulence factors, and evaluated any potential antimicrobial resistance harbored by *B. bronchiseptica* isolates obtained from swine within the U.S.

**Results:**

While a generally high degree of genomic conservation was observed among the swine *B. bronchiseptica* isolates, genetic diversity was identified within the *fimNX* locus and among the sequence type six (ST6) isolates. The majority of *B. bronchiseptica* isolates exhibited phenotypic resistance to four antibiotic classes, however, only three antimicrobial resistance genes were identified.

**Discussion:**

Combined the data suggests that *B. bronchiseptica* isolates are not serving as a source of antimicrobial resistance gene transference in the swine production environment.

## Introduction

*Bordetella bronchiseptica* is a highly contagious bacterial respiratory pathogen with a broad host range of wild and domesticated mammals, consisting of both companion and livestock animals (Mattoo and Cherry, 2005; Brockmeier et al., 2019; Chambers et al., 2019). *B. bronchiseptica* colonization is pervasive in swine herds and causes a spectrum of clinical disease outcomes ranging from asymptomatic carriage to severe bronchopneumonia (Brockmeier et al., 2019). It is the primary etiologic agent of nonprogressive atrophic rhinitis, a mild to moderately severe, reversible condition, and it promotes colonization by toxigenic strains of *Pasteurella multocida*, producing severe, progressive atrophic rhinitis (Cross, 1962; Duncan et al., 1966b; Pedersen and Barfod, 1981; Rutter, 1983; Chanter et al., 1989; de Jong and Nielsen, 1990). In young pigs, *B. bronchiseptica* is the primary cause of severe bronchopneumonia and in older pigs *B. bronchiseptica* contributes to secondary bacterial pneumonia and/ or porcine respiratory disease complex (PRDC) (Dunne et al., 1961; Duncan et al., 1966a; Brockmeier et al., 2002; Palzer et al., 2008; Brockmeier et al., 2019). Numerous studies have demonstrated that *B. bronchiseptica* colonization increases the ability of *Glaesserella parasuis*, *Pasteurella multocida*, and *Streptococcus suis* to colonize the respiratory tract of swine, while additionally increasing the severity of respiratory disease associated with these bacterial pathogens as well as viral pathogens including swine influenza virus (SIV), porcine reproductive and respiratory syndrome virus (PRRSV), and porcine respiratory coronavirus (PRCV) (Vecht et al., 1989; Vecht et al., 1992; Brockmeier et al., 2000; Brockmeier et al., 2001; Brockmeier, 2004; Brockmeier and Register, 2007; Brockmeier et al., 2008; Loving et al., 2010). Regardless of the clinical outcome, *B. bronchiseptica* infections universally result in long-term to life-long carriage (Goodnow, 1980; Akerley et al., 1995; Mattoo and Cherry, 2005; Nicholson et al., 2009; Nicholson et al., 2012; Nicholson et al., 2014; Nicholson et al., 2017).

The majority of *B. bronchiseptica* virulence gene expression is regulated by a two-component sensory transduction system encoded by the *bvg* locus (Nicholson, 2007; Nicholson et al., 2012; Nicholson et al., 2024). This locus contains BvgS, a histidine kinase sensor protein, and BvgA, a DNA-binding response-regulator protein. In response to a variety of environmental cues, BvgAS controls the expression of phenotypic phases transitioning between a virulent (Bvg+) phase and a non-virulent (Bvg–) mode. During the virulent Bvg^+^ phase, the BvgAS system is fully active and virulence-activated genes (vags), such as filamentous haemagglutinin (FHA), pertactin (PRN), fimbriae, dermonecrotic toxin (DNT), adenylate cyclase toxin (ACT), and a type III secretion system (T3SS), are fully expressed (Cotter and Jones, 2003; Nicholson, 2007; Nicholson et al., 2024).

The U.S. swine industry is the third largest producer of pork in the world and respiratory disease in pigs is the most important health concern for swine producers today (USDA-APHIS-VS-CEAH-NAHMS, 2017; FAS-USDA, 2024). Treating respiratory disease, specifically bacterial pneumonia, accounts for the highest use of antimicrobials given to both nursery-age and grower/ finisher-age pigs in the U.S. (USDA-APHIS-VS-CEAH-NAHMS, 2019). *B. bronchiseptica* is generally regarded as having a low prevalence of antimicrobial resistance (AMR) (Kadlec and Schwarz, 2018). However, swine harbor many bacterial species such as livestock-associated methicillin-resistant *Staphylococcus aureus* (LA-MRSA) and *Streptococcus suis* that are both regarded as reservoirs for AMR dissemination (Hau et al., 2018; Nicholson and Bayles, 2022). Thus, it is essential to evaluate the genetic diversity and AMR harbored by *B. bronchiseptica* isolates obtained from swine. Currently, there is limited publicly available genomic sequencing data for *B. bronchiseptica* isolates obtained from swine. Of the limited genomic sequences that are available, only one is derived from a swine isolate obtained within the U.S. The goals of the current study were to fill this gap by utilizing whole-genome sequencing (WGS) analysis to evaluate the sequence type (ST), genetic diversity of genes predicted to encode regulatory and virulence factors, and any potential AMR harbored by these isolates.

## Materials and methods

### *Bordetella bronchiseptica* isolates and culture conditions

A total of 137 *B. bronchiseptica* isolates obtained from across 20 states in the U.S. between 2015 and 2017 submitted from routine diagnostic cases were selected for the project (Supplementary Table S1). All isolates were either obtained from samples collected as part of previous studies or were obtained from samples submitted as part of field case investigations and did not require Institutional Animal Care and Use Committee (IACUC) approval. Frozen stocks (−80°C in 30% glycerol) of *B. bronchiseptica* isolates were streaked onto tryptic soy agar containing 5% sheep blood (Becton, Dickinson and Co. Franklin Lakes, NJ) and incubated overnight at 37°C with 5% CO_2_. Single colonies were inoculated into Lysogeny broth (LB) and grown aerobically at 37°C overnight in a shaking incubator (250 rpm). The previously characterized *B. bronchiseptica* strain KM22 (Nicholson et al., 2020) was included in the analyses (Supplementary Table S1).

### Whole-genome sequencing, assembly, and annotation

Genomic DNA extraction was extracted using High Pure PCR Template Preparation Kit (Roche Diagnostics Corp., Indianapolis, IN) from 500 μL broth cultures inoculated from a single colony and grown overnight aerobically at 37°C. The Qubit 1X dsDNA BR Assay Kit (Life Technologies, Eugene, OR) was used to determine DNA concentration. WGS assemblies for isolates were obtained using Illumina short read data. Library preparation was performed using a custom Illumina TruSeq-style protocol (Arbor Biosciences, Ann Arbor, MI) and sequenced on an Illumina NovaSeq 6,000 in 150 bp paired-end mode. The Illumina datasets were assessed for quality using FastQC^1^ and adapter trimming performed using BBduk^2^. *De novo* genome assembly was performed using SPAdes v. 3.15.4 in --careful mode (Bankevich et al., 2012). The resulting assemblies were filtered to retain only contigs greater or equal to 1,000 bp in length and annotated using the NCBI Prokaryotic Genome Annotation Pipeline (PGAP) v. 6.7 (Tatusova et al., 2016).

After the Illumina short read sequencing was completed, isolates D16-049392 (ST7) and D16-047428 (ST6) were chosen to have additional long-read sequencing performed with the goal of obtaining complete closed genome sequences. These isolates were chosen to represent each of the two sequence types identified in the swine isolate draft genome assemblies and represent different geographic locations (Kansas and Iowa, respectively). Short read Illumina data was obtained using the same library preparation and sequencing as described above. Genomic libraries for Nanopore sequencing were prepared with the SQK-RBK004 Rapid Barcoding Kit (Oxford Nanopore, Oxford, UK), following the manufacturer’s instructions. Sequencing was performed using a MinION Mk1C instrument with a MIN106D flow cell (version R9). The run length was 72 h and base calling was performed using Guppy v. 6.2.11 (high-accuracy mode, minimum read length of 200 bp, minimum Q score of 9). Long read data was assembled using Flye v. 2.9.1 with settings --nano-raw -g 5.3 m (Kolmogorov et al., 2019) followed by error-correction with Medaka v. 1.11.1 (Oxford Nanopore, Oxford, UK). This resulted in a single closed circular chromosome for both isolates. The Illumina short read data was then used to polish the long read assemblies using Polypolish v. 0.5.0 and POLCA v. 4.1.0 (Wick and Holt, 2022; Zimin and Salzberg, 2020, p. 208). The resulting assemblies were rotated to start at the *dnaA* gene and annotated using the NCBI Prokaryotic Genome Annotation Pipeline (PGAP) v. 6.7 (Tatusova et al., 2016). Unless otherwise specified, default software settings were used.

### Comparative genomic analysis

Multi-locus sequence typing (MLST) was performed *in silico* utilizing the BIGSdb-Pasteur databases hosted by the Institut Pasteur^3^. Average nucleotide identity (ANI) values were calculated using FastANI v. 1.33 (Jain et al., 2018), which uses a MinHash mapping-based algorithm to calculate pairwise genome-to-genome ANI values. In addition to the genome assemblies from the current study, 11 additional genomes downloaded from NCBI were included in the comparisons and are listed in Supplementary Table S1 (Parkhill et al., 2003; Okada et al., 2014; Register et al., 2015; Nicholson et al., 2020). The additional genomes included 10 swine isolates, which were publicly available assemblies in the NCBI RefSeq Database as of September 2024. Virulence-associated genes were identified by BLASTN (Altschul et al., 1997) searches and the percent identity for each gene relative to a reference gene from *B. bronchiseptica* strain KM22 was determined. Further curation for determination of a gene designation was not present, not found, or incomplete within a genome assembly was performed in Geneious Prime 2023.0.1^4^. Hierarchical clustering by isolate utilizing a complete linkage method based on Euclidean distance was performed in R using the ComplexHeatmap package (Gu et al., 2016). An *in silico* PCR based on PCR typing schemes described by Buboltz et al. was used to screen genomes for known O-antigen type O1 or O2 (Buboltz et al., 2009). To determine the presence of the *cya* or *ptp* loci, *in silico* PCR was performed in Geneious Prime 2023.0.1 (see footnote 4) using the primer sets described in Buboltz et al. (2008) to identify the respective operons. Comparison of genes encoding fimbrial protein subunits within the *fimNX* region was performed by BLASTN search (Altschul et al., 1997) to identify the region and MAFFT alignment (Katoh and Standley, 2013) to categorize the gene families.

### Phenotypic and genomic AMR analysis

Phenotypic antibiotic resistance was determined using the broth microdilution method by National Veterinary Services Laboratories (Ames, IA) following standard operating procedures. Minimum inhibitory concentrations (MICs) were determined for each isolate using the Trek BOPO7F plate (Thermo Fisher Scientific Inc., Oakwood Village, OH) with *Escherichia coli* ATCC 25922 (ATCC, Manassas, VA) serving as the quality control strain. MICs were evaluated in accordance with Clinical Laboratory Standards Institute (CLSI) recommendations based on the VET09 and M100 standards for resistance interpretations after incubation for 24 h (Pruller et al., 2015; CLSI, 2024a,b). An *in silico* search for antimicrobial resistance (AMR) genes was performed using AMRFinderPlus v. 3.12.8 with database version 2024-05-02.2 (Feldgarden et al., 2019). The input option --nucleotide was used to analyze the assembled genome FASTA sequences with default settings. Result interpretation breakpoints used for *B. bronchiseptica* were values provided by the CLSI guidelines when available (CLSI, 2024a,b). Since breakpoints specific to *B. bronchiseptica* are limited, breakpoints used for treating any infections in dogs and humans caused by *Staphylococcus* spp. or *Streptococcus* spp. were used for clindamycin, breakpoints used for treating respiratory infections in swine caused by *Actinobacillus* spp. were used for gentamycin and tiamulin, and breakpoints used for treating respiratory infections in swine and/ or cattle caused by *Pasteurella multocida* and/or *Mannheimia haemolytica* were used for penicillin, ceftiofur, tetracycline, gamithromycin, neomycin, spectinomycin, danofloxacin, enrofloxacin, tilmicosin (CLSI, 2024a). Isolates were considered resistant to sulfadimethoxine when MIC was equal to or exceeded 256 μg/mL, and isolates were considered resistant to trimethoprim / sulfamethoxazole when MIC exceeded 2 μg/mL (Vilaro et al., 2023). No interpretation breakpoints were available for tylosin. Antimicrobial susceptibility data (AST), along with test ranges and clinical breakpoints used interpretations, for all isolates are listed in Supplementary Table S2. Comparison of the association of phenotypic resistance between isolates harboring the *sul2* gene and not harboring the *sul2* gene was performed by Fisher’s exact test a using GraphPad Prism v 10.1.0 (GraphPad Software, La Jolla, CA) and *p* < 0.05 was considered significant.

### Data availability

The genome assemblies and sequencing read data have been deposited at DDBJ/ENA/GenBank under BioProject accession number PRJNA1079785. The sequencing data has been deposited in the Sequence Read Archive (SRA) under the following accession numbers SRP222122 and SRP520970. Genbank accession number for the pBORD-sul2 plasmid is PQ352461. Detailed information regarding assembly statistics, BioSample, GenBank, and SRA accession numbers is provided in Supplementary Table S1.

## Results

### Sequence type (ST) distribution

MLST was performed to begin characterizing the *B. bronchiseptica* isolates obtained from swine from across the U.S. Only two sequence types (STs) were identified with ST7 observed as the most prevalent, accounting for 95% (*n* = 130) of the isolates analyzed (Supplementary Table S1). Seven isolates were identified as ST6, accounting for 5% of the isolates (Supplementary Table S1). The ST6 isolates were not from a similar geographical location or year of isolation (Supplementary Table S1). Based on the STs, all the *B. bronchiseptica* isolates group into lineage I-1 based on the phylogenetic tree from the cgMLST-based typing method developed by Bridel et al. (2022).

### Average nucleotide identity (ANI) distribution

Whole genome ANI values were compared among the *B. bronchiseptica* swine isolates from the current study, all *B. bronchiseptica* swine isolates previously deposited in the NCBI RefSeq database (*n* = 10), and the commonly used laboratory reference strain RB50, which was isolated from a rabbit (Table 1). The STs of the isolates obtained from NCBI were ST7 (*n* = 9; swine), ST6 (*n* = 1; swine), and ST12 (*n* = 1; rabbit, RB50) (Table 1). The means of pairwise ANI values obtained when comparing the ST7 isolate D16-049392 to all other isolates from the current study and to all selected isolates sourced from NCBI were all greater than 99 (Table 1). Similarly, the means of pairwise ANI values obtained when comparing the ST6 isolate D16-047428 to all other isolates from the current study and to all selected isolates sourced from NCBI were also all greater than 99 (Table 1). The highest ANI values were observed among isolates with the same ST for every comparison (Table 1). Specifically, the mean of pairwise ANI values obtained when comparing the ST7 isolate D16-049392 to other ST7 isolates within the current study was 99.91 (Table 1). In contrast, the mean of pairwise ANI values obtained when comparing the ST7 isolate D16-049392 to other ST6 isolates within the current study was 99.76, which was slightly lower than the ANI values obtained from comparing among isolates of the same ST (ST7) (Table 1). The same trend of higher ANI values attained among isolates with the same ST is observed for all comparisons (Table 1).

**Table 1:** Pairwise ANI values calculated from comparing all isolates from the current study and selected isolates sourced from NCBI.

	Mean ANI^a^	StdDev of ANI^a^	Min ANI^a^	Max ANI^a^
D16-049392 (ST7)				
Current study: all	99.90	0.0645	99.6371	100
ST 6	99.76	0.0801	99.6371	99.8514
ST 7	99.91	0.0543	99.7245	100
NCBI: all selected	99.90	0.1071	99.5941	99.9749
ST 6	99.84	NA	99.8407	99.8407
ST 7	99.94	0.0196	99.9115	99.9749
ST 12	99.59	NA	99.5941	99.5941
D16-047428 (ST6)				
Current study: all	99.80	0.0537	99.6307	100
ST 6	99.90	0.0651	99.8073	100
ST 7	99.79	0.0465	99.6307	99.8576
NCBI: all selected	99.81	0.0923	99.5737	99.9745
ST 6	99.97	NA	99.9745	99.9745
ST 7	99.82	0.0164	99.7864	99.8412
ST 12	99.57	NA	99.5737	99.5737
KM22 (ST7)				
Current study: all	99.90	0.0578	99.6923	99.9692
ST 6	99.79	0.0577	99.6923	99.8506
ST 7	99.90	0.0515	99.7199	99.9692
NCBI: all selected				
ST 6	99.84	NA	99.8448	99.8448
ST 7	99.94	0.0284	99.8944	100
ST 12	99.59	NA	99.5875	99.5875
RB50 (ST12)				
Current study: all	99.52	0.0538	99.3284	99.6017
ST 6	99.53	0.0541	99.4612	99.5937
ST 7	99.52	0.0539	99.3284	99.6017
NCBI: all selected	99.60	0.1376	99.4913	100
ST 6	99.58	NA	99.578	99.578
ST 7	99.55	0.0329	99.4913	99.588
ST 12	100	NA	100	100

^a^Mean ANI values, standard deviation, minimum ANI values, and maximum ANI values of pairwise ANI comparisons of closed assemblies of isolates D16-049392 (ST7), D16-047428 (ST6), KM22 (ST7), and RB50 (ST12) to all assemblies from the current study (*n* = 137), ST6 assemblies from the current study (*n* = 7), ST7 assemblies from the current study (*n* = 130), all selected NCBI assemblies (*n* = 11), ST6 NCBI swine assembly (*n* = 1), ST7 NCBI swine assemblies (*n* = 9), and RB50 (ST12) NCBI assembly (*n* = 1).

The lowest ANI values were observed when comparing the ST12 laboratory reference strain RB50 to all other isolates from the current study and to all selected isolates sourced from NCBI (Table 1). However, despite these comparisons resulting in the lowest observed ANI values, the mean values were greater than 99 (means of 99.52–99.58), indicating a high degree of sequence similarity among all compared genomes (Table 1). The low standard variation of pairwise ANI values both within the current study and compared to previously sequenced swine isolates from five countries (USA, Hungary, the Netherlands, Japan, and China) and isolation dates ranging from 1988 to 2022 indicates a low genetic diversity among *B. bronchiseptica* isolates obtained from swine.

### AMR distribution

Phenotypic antimicrobial resistance was determined, and the majority of the isolates were resistant to four out of the eight antibiotic classes tested (Table 2 and Supplementary Table S2). The highest frequencies of resistance were observed for *β*-lactams, both penicillin and cephalosporin (100%, *n* = 137), macrolide/lincosamide/streptogramin (MLSb) (100%, *n* = 137), sulfonamide (100%, *n* = 137), and pleuromutilin (99%, *n* = 135) antibiotic classes (Table 2 and Supplementary Table S2). In contrast, the lowest frequencies of resistance were observed for tetracycline (0%, *n* = 0), aminoglycoside (0%, *n* = 0), and phenicol (<1%, *n* = 1) antibiotic classes (Table 2 and Supplementary Table S2).

**Table 2:** Phenotypic AMR prevalence among swine *B. bronchiseptica* isolates.

Antibiotic class	Antibiotic	Number (%) ^a^
β-lactam/Penicillin	Ampicillin	137 (100%)
	Penicillin	137 (100%)
β-lactam/Cephalosporin	Ceftiofur	137 (100%)
Tetracycline	Tetracycline	0 (0%)
Macrolide/Lincosamide/Streptogramin (MLSb)	Clindamycin	137 (100%)
	Gamithromycin	0 (0%)
	Tilmicosin	3 (2%)
	Tildipirosin	0 (0%)
	Tulathromycin	0 (0%)
	Tylosin	0 (0%)
Aminoglycoside	Gentamicin	0 (0%)
	Neomycin	0 (0%)
	Spectinomycin	0 (0%)
Phenicol	Florfenicol	1 (<1%)
Fluroquinolone	Danofloxacin	9 (7%)
	Enrofloxacin	1 (<1%)
Sulfonamide	Sulfadimethoxine	137 (100%)
	Trimethoprim/Sulfamethoxazole	5 (4%)
Pleuromutilin	Tiamulin	135 (99%)

^a^ Number of isolates resistant to indicated antibiotic (percentage of isolates tested).

Focusing on specific antibiotics tested, no isolates were found to be phenotypically resistant to the following antibiotics: tetracycline, gamithromycin, tildipirosin, tulathromycin, gentamicin, and neomycin (Table 2). One hundred and thirteen isolates (83%) exhibited intermediate resistance to danofloxacin, and 77 isolates (57%) exhibited intermediate resistance to enrofloxacin (Supplementary Table S2). Although specific breakpoints are unavailable for tylosin, all isolates tested except one exhibited high MIC values of equal to or greater than 32 μg/mL (Supplementary Table S2). Resistance to sulphadimethoxine was tested only at a MIC of 256 μg/mL and all isolates exhibited a MIC equal to or greater than 256 μg/mL and were considered resistant (Supplementary Table S2). Five isolates exhibited resistance to trimethoprim / sulfamethoxazole (Supplementary Table S2). The interpretation breakpoints used for spectinomycin were MIC values less than or equal to 32 μg/mL were considered susceptible, MIC values equal to 64 μg/mL were considered intermediate, and MIC values greater than or equal to 128 μg/mL were considered resistant (Supplementary Table S2). The highest MIC value tested for spectinomycin was 64 μg/mL (Supplementary Table S2). Given that a MIC value of >64 μg/mL was observed for all isolates, which is above the intermediate interpretation breakpoint MIC value and the resistant MIC value was not tested, no interpretation was used to classify the isolates (Supplementary Table S2).

Genomes were screened for chromosomal mutations and genes conferring AMR and three AMR genes, the *Bordetella*-specific *β*-lactamase gene *blaBOR* (Lartigue et al., 2005), the sulfonamide resistance gene *sul2*, and the aminoglycoside resistance gene *aph(3″)-Ib* or *strA*, were identified among the *B. bronchiseptica* swine isolates (Supplementary Table S2). All of the swine isolates harbored the β-lactamase gene *blaBOR* (Supplementary Table S2). The sulfonamide resistance gene *sul2* was also highly prevalent and identified in 128 of the 137 isolates analyzed (Supplementary Table S2). Additionally, a statistically significant association was detected between sulfonamide resistance and the presence of the *sul2* gene among the analyzed isolates (*p* = 0.0002). In contrast the aminoglycoside resistance gene *aph(3″)-Ib* or *strA* was harbored by only one isolate, D17-015854 (Supplementary Table S2). While the isolate harboring the *aph(3″)-Ib* gene did not exhibit resistance to any aminoglycoside class of antibiotics tested, the isolate did exhibit intermediate resistance to spectinomycin (Supplementary Table S2).

**Figure 1:**
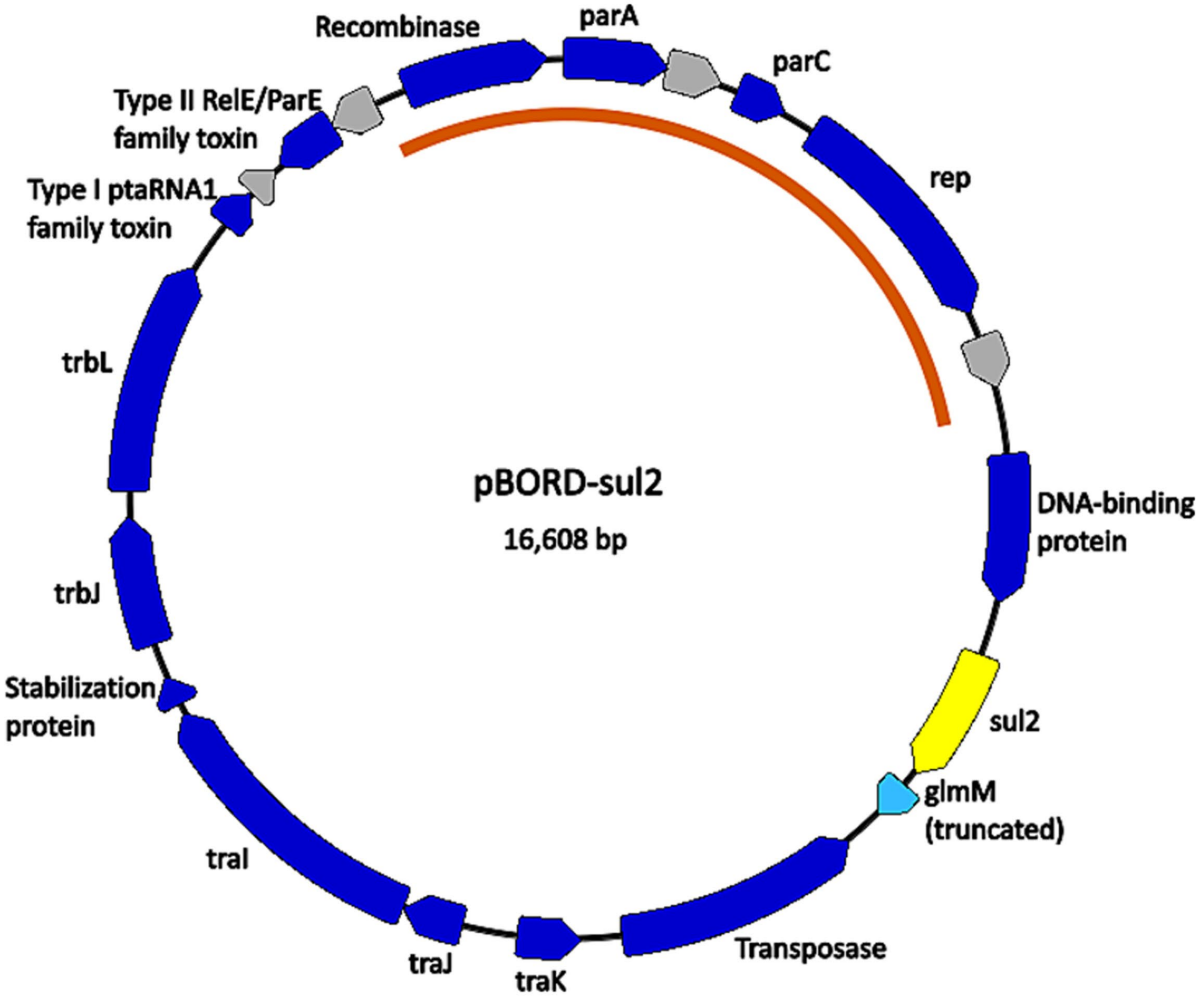
Map of the plasmid found in *B. bronchiseptica* swine isolates D16-039234, D17-011401, and D17-019744. Nucleotide sequences for plasmid pBORD-sul2 (accession #PQ352461) correspond to contig 33 for D16-039234, contig 92 for D17-011401, and contig 79 for D17-019744. Dark blue arrows are annotated CDSs with predicted functions. Grey arrows are CDSs with unknown function, annotated by PGAP as “hypothetical protein CDS.” The light blue arrow is a predicted pseudogene (truncated CDS). The yellow arrow is the *sul2* antimicrobial resistance gene. The orange arc indicates the portion of the plasmid sequence highly conserved with *B. bronchiseptica* plasmid pKBB4037 (accession # AJ877266.1).

The chromosomal location of the *aph(3″)-Ib* gene was investigated further in isolate D17-015854 and a gene predicted to encode a recombinase followed by a gene predicted to encode a Tn3 family transposase gene were identified directly next to the *aph(3″)-Ib* gene, indicating that the chromosomal region could possibly be a mobile element. The chromosomal location of the *sul2* gene was also investigated and a predicted transposase gene of the IS91-like element ISVsa3 family transposase was identified along with predicted mobile element related genes, recombinase/integrase and conjugation related genes were identified in close proximity to the *sul2* gene in the majority of the isolates that harbored *sul2*. The close proximity of the transposase suggests that the chromosomal region in these isolates could possibly be a mobile element. In contrast, for three isolates, D16-039234, D17-011401, and D17-019744, the *sul2* gene was located on a contig of approximately 16 kb in size that also contained the plasmid replication genes *parA* and *parC*. Further analyses revealed that the ends of the contig sequence overlap and resulted in a circularize plasmid sequence that was identical among all three isolates (Figure 1). The plasmid was named pBORD-sul2 (accession number PQ352461) and harbors an approximately 5 kb region containing the *rep*, *parA,* and *parC* genes with 99.47% nucleotide sequence identity to a previously reported 11 kb *B. bronchiseptica* plasmid pKBB4037 harboring *tetA* gene (Kadlec et al., 2006) (Figure 1).

### Diversity of regulatory and virulence-associated genes

**Figure 2:**
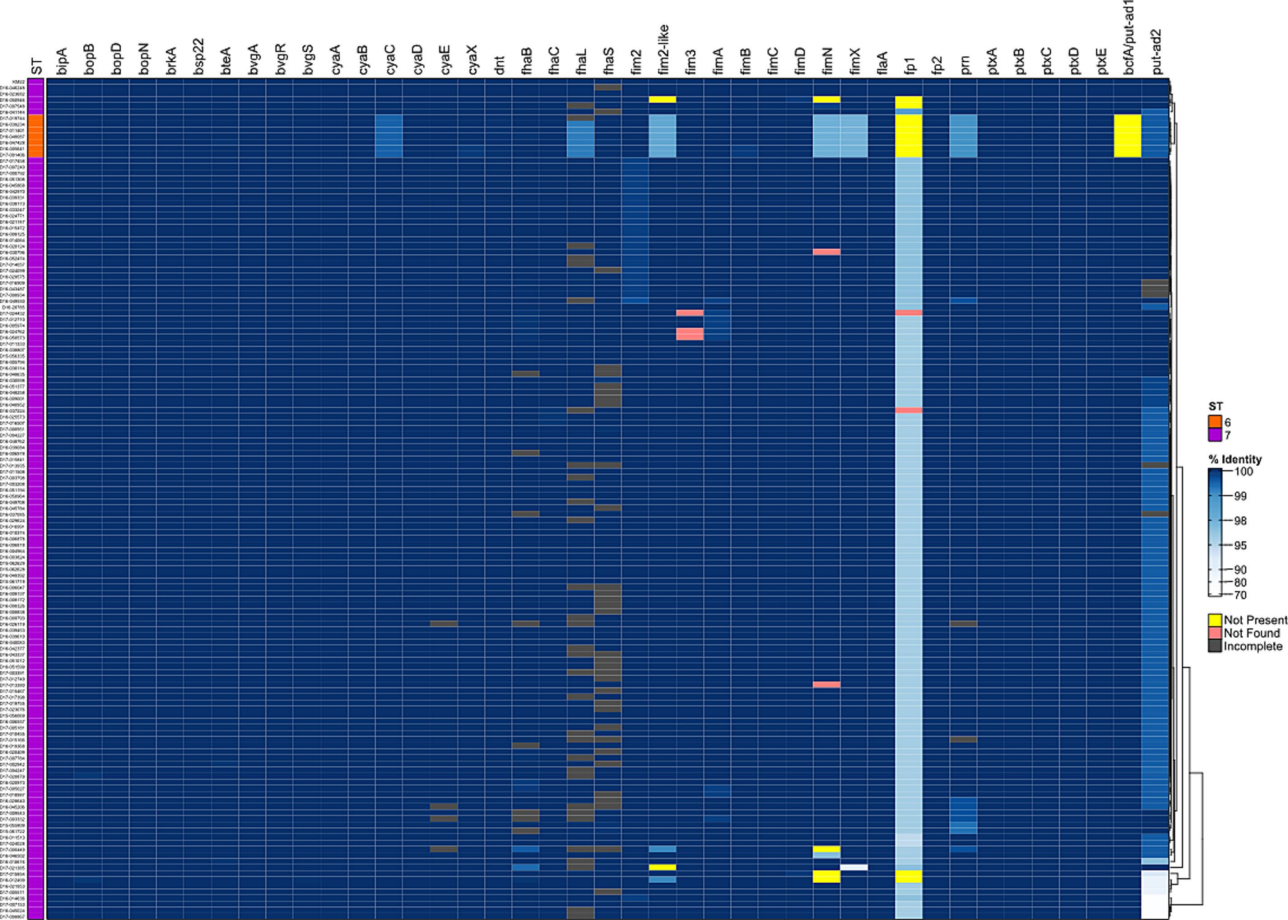
Hierarchical cluster heatmap displaying the relatedness of *B. bronchiseptica* swine isolates based on the nucleotide percent identity of analyzed virulence genes. Nucleotide percentage identity values for analyzed genes were used to generate a distance matrix heatmap clustered by hierarchical clustering using a complete linkage method with Euclidean distance. Gene names are provided at the top of heatmap, and isolate names are provided at the left side of heatmap. Nucleotide percentage identity is represented using the color scale shown to the right side of heatmap. ST is provided at the left side of heatmap and is represented using the colors shown left of heatmap. Genes designated as not present, not found, or incomplete are represented using the colors shown right of heatmap. Dendrogram is on the right side of the heat map.

To further examine genomic diversity among the *B. bronchiseptica* swine isolates, we compared the nucleotide sequences of genes encoding well-characterized regulatory factors and virulence factors. The percent identity for each gene was determined for each isolate relative to the KM22 orthologue. Overall, a high degree of nucleotide sequence identity was observed for all analyzed genes encoding regulatory and virulence factors. The nucleotide sequence identity for regulatory genes *bvgA* and *bvgR* was 100%, the identity for *bvgS* ranged from 99.97 to 100% (Supplementary Table S3). With the exception of predicted fimbrial and adhesin genes, the nucleotide sequence identity for all other genes encoding well-characterized virulence factors were highly conserved and ranged from 99.22 to 100% with lower identity observed for *cyaC*, *prn*, and *fhaL* genes among ST6 isolates (Figure 2 and Supplementary Table S3).

It’s has been previously demonstrated that the *cya* operon, comprising genes that encode, activate, and the secrete adenylate cyclase toxin, was replaced by an operon predicted to encode peptide transport (*ptp*) proteins in *B. bronchiseptica* ST37 isolates (Buboltz et al., 2008). Genes *cyaA*, *cyaB*, *cyaC*, *cyaD*, and *cyaE* of the *cya* operon were all present and highly conserved among the *B. bronchiseptica* swine isolates analyzed (Figure 2 and Supplementary Table S3).

The *wbm* locus contains genes required for expression of three antigenically distinct O-antigen types defined as O1- or O2- or O3 serotype (Preston et al., 1999; Buboltz et al., 2009; Vinogradov et al., 2010; Hester et al., 2013). An *in silico* PCR based on PCR typing schemes described by Buboltz et al. was used to screen the genomes of all isolates (Buboltz et al., 2009). Similar to KM22, all swine isolates harbored a *wbm* locus encoding genes for an O-antigen serotype O2 (data not shown).

The greatest nucleotide sequence divergence was observed for predicted fimbrial and adhesin genes *putative-adhesin 2* (*put-ad2*), *fimbrial protein 1*, *fimN*, and *fimX* (Figure 2 and Supplementary Table S3). The *putative-adhesin 2* (*put-ad2*) gene was the most divergent of all genes analyzed with nucleotide sequence identity that ranged from 72.34 to 100% (Figure 2 and Supplementary Table S3). The nucleotide sequence identity for *fimbrial protein 1* ranged from 94.29 to 100% (Figure 2 and Supplementary Table S3). The nucleotide sequence identity for *fimN* ranged from 96.86 to 100% and *fimX* ranged from 89.34 to 100% (Figure 2 and Supplementary Table S3).

In addition to having lower nucleotide sequence identity compared to all other virulence-associated genes analyzed, the predicted fimbrial and adhesin genes *fimN*, *fim2*, *fimbrial protein 1*, and *bcfA*/*putative adhesin 1* (*bcfA*) were absent from the genomes of some isolates. The absence of *bcfA* and *fimbrial protein 1* genes from ST6 isolates were the most notable (Figure 2 and Supplementary Table S3). The *bcfA* gene was absent from genomes of all seven ST6 isolates (Figure 2 and Supplementary Table S3). The *fimbrial protein 1* gene was absent from genomes of eleven isolates, four ST7 isolates and all seven ST6 isolates (Figure 1 and Supplementary Table S2). The *fimN* gene was absent from genomes of four ST7 isolates and the *fim2* gene was absent from genomes of two ST7 isolates (Figure 2 and Supplementary Table S3).

**Figure 3:**
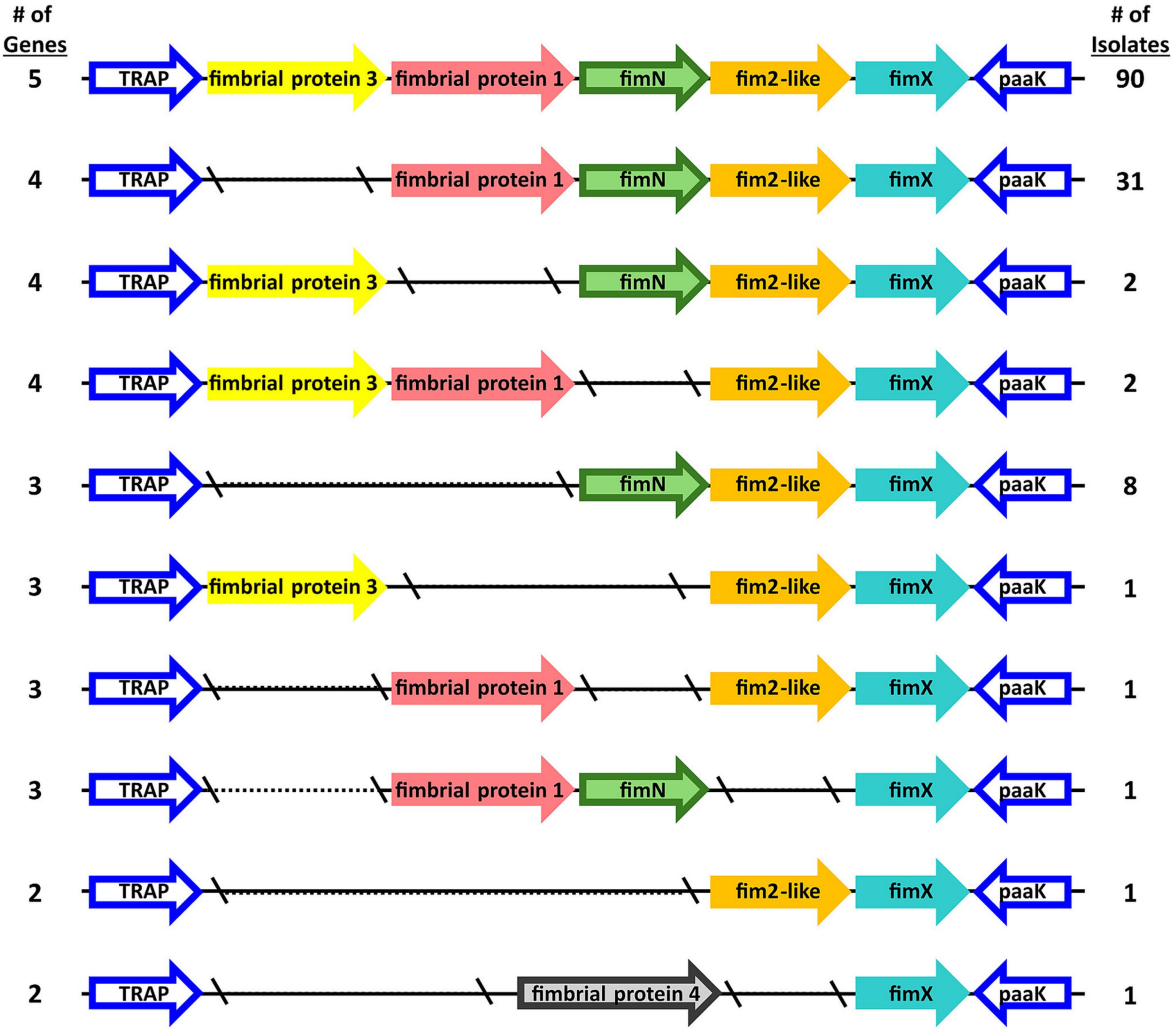
Organization of *fimNX* locus. Predicted fimbrial protein genes are represented as arrows. Gene names refer to names used in Supplementary Table S4 and color-coded by gene family, which is defined by nucleotide sequence identity.

Further focusing on the diversity within the *fimNX* locus, the number of genes harbored within this locus varied from two to five among the *B. bronchiseptica* swine isolates (Figure 2 and Supplementary Table S4). In addition to the number of number of genes harbored within this locus, the type of predicted fimbrial genes located within this locus also varied among the *B. bronchiseptica* swine isolates (Figure 3 and Supplementary Table S4). Despite the diversity in the number and type of predicted fimbrial genes harbored, the *fimNX* locus was located in the same genomic location flanked by tripartite ATP-independent periplasmic (TRAP) transporter and phenylacetate-CoA ligase gene (*paaK*) genes in all swine isolates and in KM22 (Figure 3 and Supplementary Table S4).

Ninety swine isolates were observed to harbor five predicted fimbrial genes within the *fimNX* locus. The five predicted fimbrial genes harbored by these isolates were *fimbrial protein 3*, *fimbrial protein 1*, *fimN*, *fim2-like* and *fimX* (Figure 3 and Supplementary Table S4). The predicted fimbrial gene *fimbrial protein 3* was originally named based on the draft annotation of KM22, which harbors four predicted fimbrial genes within this locus: *fimbrial protein 1*, *fimN*, *fim2-like* and *fimX* (Figure 3 and Supplementary Table S4) (Nicholson et al., 2016). An orthologous gene to RB50 fimbrial protein gene BB3193 was identified and located in a different chromosomal location in the draft annotation of KM22 and named *fimbrial protein 2* (locus_tag KM22_03128) (Nicholson et al., 2016). The locus_tag was subsequently changed to CJ015_08855 in the closed KM22 genome annotation (Nicholson et al., 2020). Since the name “*fimbrial protein 2”* was assigned to the predicted fimbrial protein gene (locus_tag CJ015_08855) located in a different chromosomal location, the name *fimbrial protein 3* was assigned to the additional predicted fimbrial gene located in the *fimNX* locus of the 90 swine isolates that were observed to harbor it (Figure 3 and Supplementary Table S4).

Thirty-one swine isolates, including KM22, harbored four predicted fimbrial genes (*fimbrial protein 1*, *fimN*, *fim2-like* and *fimX*) within the *fimNX* locus (Figure 3 and Supplementary Table S4). Four other swine isolates also harbored four predicted fimbrial genes, but different types of predicted fimbrial genes were observed within the *fimNX* locus (Figure 3 and Supplementary Table S4). Two isolates harbored *fimbrial protein 3*, *fimN, fim2-like* and *fimX*, while two other isolates harbored *fimbrial protein* 3, *fimbrial protein 1*, *fim2-like* and *fimX* (Figure 3 and Supplementary Table S4). Eight swine isolates harbored three predicted fimbrial genes, which included *fimN, fim2-like* and *fimX* (Figure 3 and Supplementary Table S4). One isolate harbored three predicted fimbrial genes, which included *fimbrial protein 3*, *fim2-like* and *fimX* (Figure 3 and Supplementary Table S4). One isolate also harbored three predicted fimbrial genes, which included *fimbrial protein 1*, *fim2-like* and *fimX*, and another isolate was also observed to harbor three predicted fimbrial genes, which included *fimbrial protein 1*, *fimN,* and *fimX* (Figure 3 and Supplementary Table S4). Two swine isolates harbored two predicted fimbrial genes within the *fimNX* locus. One isolate harbored *fim2-like* and *fimX*, while the other isolate harbored *fimbrial protein 4* and *fimX* (Figure 3 and Supplementary Table S4). The gene *fimbrial protein 4* was annotated as a newly identified predicted fimbrial gene within the *fimNX* locus due to its low nucleotide sequence identity to the other known predicted fimbrial genes (Figure 3 and Supplementary Table S4).

## Discussion

An overall high degree of genomic conservation was observed among the swine *B. bronchiseptica* isolates analyzed despite the wide variation in geographical location, encompassing 20 different states within the U.S., and time frame in which the *B. bronchiseptica* isolates were acquired. Specifically, out of 137 isolates analyzed, only two STs were identified, ST6 and ST7. ST7 accounted for 95% (*n* = 130) of the isolates analyzed. Additionally, the high ANI values combined with the low standard variation attained by comparing all isolates from the current study, along with selected isolates sourced from NCBI from five countries and isolation dates ranging from 1988 to 2022, indicates an extremely low genetic diversity among *B. bronchiseptica* isolates obtained from swine. Apart from fimbrial and adhesin genes, the nucleotide sequence identity for all other genes encoding well-characterized regulators and virulence factors were highly conserved and ranged from 99.22 to 100%. Additionally, every *B. bronchiseptica* swine isolate harbored a *wbm* locus encoding genes for the same O-antigen serotype O2.

When evaluating the genes encoding regulators and virulence factors, the most genetic diversity observed was among the ST6 isolates and within the *fimNX* locus. The diversity observed for ST6 isolates included the lowest nucleotide sequence identity detected for *cyaC*, *prn*, and *fhaL* genes among ST6 isolates compared to other isolates and ST6 isolates additionally did not harbor genes *bcfA* and *fimbrial protein 1*. Only two other genome sequences are available for ST6 *B. bronchiseptica* isolates. These isolates are S798 (accession # GCA_000829175.1), isolated from a pig in Japan in 1988, and MBORD731 (accession # GCA_000698985.1), isolated from a horse in Denmark with no isolation date provided. Similar to the ST6 isolates in this study, both S798 and MBORD731 do not harbor genes *bcfA* and *fimbrial protein 1*. Due to the low number of ST6 *B. bronchiseptica* isolates available for examination so far, it is unclear if the absence of these genes is specific to ST6 isolates. The diversity observed within the *fimNX* locus includes both the number of genes harbored within this locus and the type of predicted fimbrial genes located within this locus. Additionally, a newly identified predicted fimbrial gene was detected within the *fimNX* locus of one of the swine *B. bronchiseptica* isolates.

A generally regarded universal trait of *B. bronchiseptica* isolates is that they tend to be resistant to *β*-lactam, both penicillins and cephalosporins, and macrolide antibiotic classes (Woolfrey and Moody, 1991; Mattoo and Cherry, 2005; Kadlec and Schwarz, 2018; Reagan, 2021). While information on the genetic basis of these resistances is sparse, low permeability to cephalosporins as well as the *Bordetella*-specific β-lactamase gene *blaBOR* have been shown to contribute to resistance to the β-lactam classes (Lartigue et al., 2005; Kadlec et al., 2007). Prior to the emergence of macrolide-resistant *B. pertussis* isolates, macrolide resistance was an important clinical distinction between *B. pertussis* and *B. bronchiseptica* (Woolfrey and Moody, 1991; Mattoo and Cherry, 2005). When the swine *B. bronchiseptica* isolates were evaluated for phenotypic antimicrobial resistance, most of the isolates were resistant to, β-lactam, macrolide, and pleuromutilin antibiotic classes. Prior susceptibility testing has reported high MIC values for pleuromutilins, specifically tiamulin (Pruller et al., 2015). In swine, tiamulin is commonly used to treat dysentery caused by *Brachyspira hyodysenteriae* and bacterial pneumonia caused by *Actinobacillus pleuropneumoniae* and/or *Mycoplasma hyopneumoniae* (Maes et al., 2020). While no pleuromutilin-inactivating enzymes have been described to date, collateral sensitivity from lincosamide genes *lnuB* and *lsaE* have been reported to confer resistance to pleuromutilins (Sharkey et al., 2016; Hawkins et al., 2017). However, none of the *B. bronchiseptica* isolates examined in this study harbored the lincosamide genes *lnuB* or *lsaE*. In contrast, the *Bordetella*-specific β-lactamase gene *blaBOR* (Lartigue et al., 2005), was found in all *B. bronchiseptica* isolates examined in this study, which is likely the genetic basis underpinning the observed β-lactam resistance. It is worth noting that the classification of phenotypic resistance based on clinical breakpoints used in this study has some limitations, such as increasing the difficulty in associating phenotypic resistance to genotypic resistance mechanisms.

When the genomes of the *B. bronchiseptica* isolates were screened for AMR elements, only three AMR genes were identified. As previously mentioned, the Bordetella-specific β-lactamase gene *blaBOR* (Lartigue et al., 2005) was found in all *B. bronchiseptica* isolates examined in this study. Many of the isolates were also found to harbor the sulfonamide resistance gene *sul2*, and a statistically significant association was detected between sulfonamide resistance and the presence of the *sul2* gene. Previous studies have described co-selection due observed linkage of aminoglycoside resistance gene *aph(3″)-Ib* or *strA* and sulfonamide resistance genes (Kehrenberg and Schwarz, 2001; Pruller et al., 2015). However, the *B. bronchiseptica* isolate harboring the *aph(3″)-Ib* or *strA* gene did not harbor the *sul2* gene. Predicted transposase genes were identified located in close proximity to the *aph(3″)-Ib* or *strA* gene and the *sul2* gene, for the isolates that harbored a chromosomally located *sul2* gene. The close proximity of the predicted transposases indicated that those genomic regions could possibly function as mobile genetic elements (MGEs). However, no additional indicators suggesting that these regions function as MGEs were identified. Three isolates were found to harbor the *sul2* gene on an identical 16 kb plasmid. The plasmid identified among these three isolates was the only MGE carrying an AMR gene identified in this study. Given the relatively ease of transferase of bacterial plasmids, it was unexpected that either this plasmid or other similar plasmids were not found in more *B. bronchiseptica* isolates examined in this study.

Overall, very few AMR elements were identified among the swine *B. bronchiseptica* isolates examined in this study. In fact, only three isolates were identified to harbor one AMR gene on a defined MGE. Additionally, a high degree of genomic conservation of analyzed genes was observed among the swine *B. bronchiseptica* isolates analyzed. Combined, the genotypic and phenotypic data reported here for *B. bronchiseptica* isolates is in stark contrast to similar data previously reported for other bacterial species known for colonizing swine, such as LA-MRSA and *S. suis* (Hau et al., 2018; Nicholson and Bayles, 2022). Both LA-MRSA and *S. suis* are regarded as reservoirs for AMR dissemination because they are typically resistant to multiple classes of antibiotics and harbor numerous AMR genes on MGEs (Hau et al., 2018; Nicholson and Bayles, 2022).

The lack of genomic diversity observed among the swine *B. bronchiseptica* isolates, including only two STs identified, high ANI values among analyzed isolates, the high degree of sequence conservation of analyzed genes, along with the few AMR elements harbored by these isolates, indicates that *B. bronchiseptica* swine isolates are not readily sharing genes or exchanging DNA with other bacterial community members in their environment. Collectively, the data reported in this study deriving from a broad inclusion of *B. bronchiseptica* isolates obtained from across 20 states in the U.S. supports previous findings showing a low prevalence of AMR genes among *B. bronchiseptica* isolates (Kadlec and Schwarz, 2018), while also indicating that *B. bronchiseptica* is not serving as a source of antimicrobial resistance and MGEs in the swine production environment.

## Data Availability

The genome assemblies and sequencing read data have been deposited at DDBJ/ENA/GenBank under BioProject accession number PRJNA1079785. The sequencing data has been deposited in the Sequence Read Archive (SRA) under the following accession numbers SRP222122 and SRP520970. Genbank accession number for the pBORD-sul2 plasmid is PQ352461. Detailed information regarding assembly statistics, BioSample, GenBank, and SRA accession numbers is provided in Supplementary Table S1.

## Footnotes

^1^https://www.bioinformatics.babraham.ac.uk/projects/fastqc/

^2^https://sourceforge.net/projects/bbmap/

^3^https://bigsdb.pasteur.fr

^4^https://www.geneious.com
